# Using Mobile Technology for Family-Based Prevention in Families with Low Incomes: Lessons from a Randomized Controlled Trial of a Childhood Obesity Prevention Program

**DOI:** 10.1007/s11121-023-01637-8

**Published:** 2024-02-07

**Authors:** Thomas G. Power, Susan S. Baker, Karen V. Barale, M. Catalina Aragón, Jane D. Lanigan, Louise Parker, Karina Silva Garcia, Garry Auld, Nilda Micheli, Sheryl O. Hughes

**Affiliations:** 1 Washington State University, Pullman, WA USA; 2 Colorado State University, Ft. Collins, CO USA; 3 Washington State University Extension, Tacoma, WA USA; 4 Maternal and Child Health, Oregon Health Authority, Portland, OR USA; 5 Washington State University, Vancouver, WA USA; 6 Washington State University Extension, Seattle, WA USA; 7 Department of Public Health, County of San Luis Obispo, San Luis Obispo, CA USA; 8 USDA/ARS Children’s Nutrition Research Center, Baylor College of Medicine, 1100 Bates Avenue, Houston, TX 77030 USA

**Keywords:** Mobile technology, Parenting education, Feeding, Childhood obesity, EFNEP

## Abstract

Researchers are increasingly using web-based technologies to deliver family-based, prevention programming. Few studies have examined the success of such approaches for families with low incomes. The purpose of this study was to describe the level of in-class and online engagement in a childhood obesity prevention program for parents with low incomes, to examine the demographic correlates of parent engagement, and to examine dosage effects on parental feeding outcomes as a function of online exposure. All participants attended in-class nutrition education classes (Eating Smart · Being Active) as part of the Expanded Food and Nutrition Education Program (EFNEP) in Colorado and Washington State (classes were offered in English and Spanish). Participants in this analysis were 168 parents from a larger cluster randomized controlled trial who had been randomly assigned to also receive a newly developed, mobile-based version of an efficacious, feeding-focused, childhood obesity prevention program. Results showed that despite high levels of in-person attendance (70%), participants only accessed 47% of the videos (online content). Older parents and parents of girls showed higher levels of in-person attendance; currently employed parents showed lower levels. Online engagement varied as a function of ethnicity and acculturation: non-Hispanic parents accessed the most videos, low-acculturated Hispanic parents accessed the second most, and highly acculturated Hispanic parents accessed the least. In contrast, low-acculturated Hispanic parents showed the highest in-person attendance. For all but one outcome, significant online program effects were found only for parents who accessed at least half of the videos. Implications for mobile-based, family-based prevention programs for parents with low incomes are considered.

ClinicalTrials.gov Identifier: NCT03170700; Registration Date: March 08, 2017.

## Introduction

Over the last 15 years, family-based prevention programming has increasingly relied on electronic technology for program implementation—to supplement in-person classes or as the primary method of program delivery (Flujas-Contreras et al., 2019). By increasing program accessibility and reducing costs, such technology has the potential to expand the reach of prevention programming beyond participants typically reached through in-person classes (Corralejo & Rodriguez, 2018). Moreover, web-based and mobile technology allow for the tailoring of programming to the individual’s needs through remote coaching, individualized messages, progress monitoring, and personalized feedback (Hall & Bierman, 2015).

The use of technology has become popular in the childhood obesity prevention area as well (Qiu et al., 2022; Zarnowiecki et al., 2020). In a review of 119 family-based, childhood obesity prevention programs, Ash et al. (2017) found that 23% of the programs were delivered exclusively through technology and an additional 12% had both in-person and technology components. Although such approaches can expand parents’ access to and engagement in prevention programming, participant involvement in such programs is highly variable, attrition is high, and programs typically work better for more educated parents (Hall & Bierman, 2015).

Online programs may be particularly problematic for low-income populations given the digital divide in the U.S. (Harris et al., 2020). However, the divide is shrinking: in 2000, 34% of households in the U.S. with incomes < $30,000 used the internet compared to 81% of those with incomes ≥ $75,000. In 2021 these numbers were 86% and 99% respectively, with adults with low incomes relying more on smartphones for internet access (Pew Research Center, 2022).

Despite the growing use of web-based technology in family-based prevention, few evaluations of such programs have been conducted on families with low incomes. In their meta-analysis of studies examining the effectiveness of technology-assisted parenting interventions for families experiencing social disadvantage (i.e., low socioeconomic status, single parenthood, or young parenthood), Harris et al. (2020) found only nine studies published between 2007 and 2019. In the review of the literature for the current paper, we located only three more recent studies (Estrada et al., 2019; Murry et al., 2019; Rojas et al., 2021). Evaluations of technology-based *childhood obesity* prevention programs for families with low incomes are also rare. Of the family-based, childhood obesity prevention programs using web-based technology cited by Ash et al. (2017), excluding study protocol publications, none targeted families with low incomes. In two reviews of technology-based nutrition education (Gomes et al., 2021; Zarnowiecki et al., 2020), only one study of families with low incomes was cited. Similarly, in a meta-analysis of obesity programs for Hispanic youth (including both family- and youth-based programs), St. George et al. (2022) found that only 4% of programs employed technology for content delivery.

Given the limited research on web-based prevention for families with low incomes, several issues need to be addressed. First, since most U. S. families with low incomes now have internet access, is this an effective way to engage them in prevention programming? Second, do parents differ in their level of engagement and what are the predictors of engagement? Finally, does the level of parent involvement predict program outcomes? We examined these issues in a study of an online version of an efficacious, family-based childhood obesity prevention program.

Childhood obesity continues to be a significant health problem (Ayala-Marin et al., 2020). About 35% of 2- to 19-year-olds in the U.S. have overweight or obesity (Fryer et al., 2020), with higher rates in families with low incomes (Ayala-Marin et al., 2020). These rates are concerning given the consequences of obesity for children’s physical and mental health (Ayala-Marin et al., 2020). Family-based obesity prevention programs may be particularly effective for young children because parents manage those child behaviors that contribute to obesity (diet, physical activity, media use, and sleep). Many family-based prevention programs have been developed in the last 10 years (Ash et al., 2017; Pamungkas & Chamroonsawasdi, 2019).

Considerable research on children’s eating behavior shows that *how* parents feed their children may be as important as *what* they feed them in contributing to obesity risk (Yee et al., 2017). Specifically, parental feeding practices can promote children’s self-regulation of caloric intake and help reduce overeating and later obesity (Grammer et al., 2022). Although eating-focused, family-based obesity prevention programs typically provide nutrition education (Ash et al., 2017; Pamungkas & Chamroonsawasdi, 2019), some recent programs (e.g., Eneli et al., 2015; Nix et al., 2021) promote eating self-regulation by encouraging parental feeding practices that are responsive to children’s cues of hunger and fullness (Grammer et al., 2022). Parents in such programs are taught to promote eating self-regulation by providing a healthy food environment, teaching children to be responsive to their internal cues of hunger and fullness, and not override these cues with excessive parental control or indulgent feeding.

One of the first family-based, obesity prevention programs with a self-regulation focus was Strategies for Effective Eating Development (SEEDS) (Hughes et al., 2016). In this program, mothers and their preschool children attend seven weekly group sessions. While parent educators teach mothers responsive feeding practices and strategies to get their children to try new foods, early childhood educators work with children to encourage exploring and trying new foods and attending to internal cues of hunger and fullness. Mothers and children also come together for a family session reinforcing the session’s content. A cluster randomized controlled trial (RCT) (Hughes et al., 2021) of the program with 255 Hispanic mothers with low incomes showed that 12 months after the final session, mothers in the prevention group, compared to controls, reported more feeding practices that promote the trying of new foods and encourage eating self-regulation, and their children were less likely to have overweight or obesity.

Given the success of SEEDS, Hughes et al. (2020) developed an online version of the program for parents (Food, Feeding, and Your Family—FFYF) and evaluated it by pairing it with a well-validated, in-person healthy eating, active living curriculum for parents of young children—Eating Smart · Being Active (ESBA) (Auld et al., 2015). In a cluster RCT, parents with low incomes who were enrolled in the Expanded Food and Nutrition Education Program (EFNEP—USDA, 2017) in Colorado and Washington State were assigned to one of three conditions: online (in-person ESBA plus a mobile phone-based version of FFYF), in-class (in-person ESBA plus an in-person version of FFYF), and control (in-person ESBA alone). Pre- to posttest analyses showed that parents in the in-class and online conditions (compared to controls) showed numerous changes in their feeding knowledge and practices (Hughes et al., 2022). The present analyses examined data from the online group to describe participant engagement, identify demographic predictors of engagement, and examine dosage effects (online exposure) on feeding outcomes.

Given the limited amount of previous research and the rapidly changing nature of online technologies, it is difficult to draw firm conclusions about the typical level of engagement in such interventions by parents with low incomes. Many early studies provided parents with free access to technology for use in the intervention (e.g., 5 of the 9 studies in the Harris et al., 2020 review), raising the possibility that the novelty of technology in the home may have inflated levels of parent engagement. Three recent studies of web-based parenting programs offer some clues. In these studies, participants were responsible for accessing the program over multiple weeks on their own (i.e., parents were not provided with free technology nor did groups of parents complete the program at a computer lab). In two studies targeting substance use and risky sexual behavior in Hispanic adolescents, Estrada et al. (2019) found that parents viewed a mean of 5.9 out of 8 parenting sessions (74%), whereas parents in a study by Rojas et al. (2021) viewed 5.4 out of 12 sessions (45%). Love et al. (2016) implemented a modified version of the Triple P Online parenting program (Turner & Sanders, 2013) in a high-risk, ethnically diverse sample of parents of 2- to 12-year-olds. The percentage of parents completing the 8-module program in their two cohorts was 36% and 51%. Given the results of these previous studies, we expected that parents in the current study would access 50–60% of the online content.

The second question concerned the predictors of program engagement. Previous work on *in-person* parenting programs shows lower levels of attendance and/or higher rates of attrition for younger parents, single parents, and for parents with lower incomes, lower education levels, or larger families (Robinson et al., 2016). In the only study we could find for a web-based program in parents with low incomes, Perrino et al. (2018), in an analysis of the data from the online Estrada et al. (2019) study cited earlier, found that less acculturated Hispanic parents showed the highest attendance levels. We therefore predicted that parents in our Spanish language classes, parents who showed low levels of acculturation, older parents, and parents with more education would show the greatest engagement in our program (i.e., attend more in-person ESBA lessons, access more of the online FFYF materials, and show lower attrition). We expected less engagement for single parents, employed parents, and parents with larger families.

Finally, we examined dosage effects on the online program outcomes. We predicted that parents with the highest levels of online engagement (i.e., accessed more online materials) would show greater effects of FFYF on their feeding knowledge and practices. We also expected that the feeding knowledge and practices of parents from groups randomly assigned to the online condition who accessed no online materials would not significantly differ from parents in groups randomly assigned to the control condition (i.e., parents who had no exposure to FFYF content).

## Methods

### Participants

**Fig. 1:**
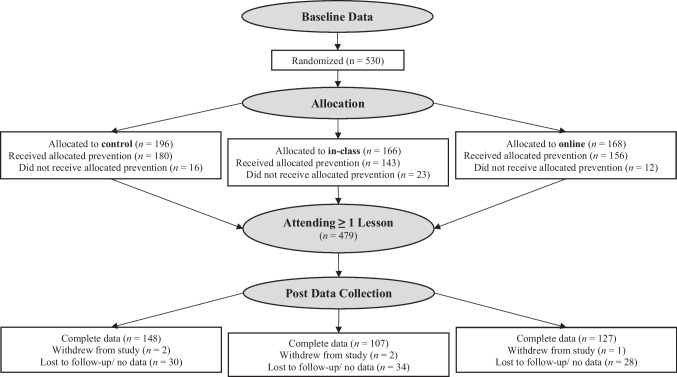
Consort flow diagram

Between September 2017 and May 2019, parents of 2- to 8-year-olds were recruited from three counties in Colorado and three counties in Washington from community agencies serving families with low incomes (see Hughes et al., 2020 for details). Classes were offered in both English and Spanish. The person primarily responsible for feeding the target child was eligible to participate if they met the following criteria: spoke English or Spanish, met EFNEP eligibility requirements, had at least one child between 2 and 8 years, had internet access, had access to a smartphone, and had not previously participated in EFNEP. A power analysis with G-Power (3.1.1), correcting for the clustering of participants within implementations (see Hughes et al., 2020), showed that a final sample of 374 parents would yield power of 0.90 to identify a small effect size (*f* = .10). Assuming a possible attrition rate of 70%, 530 parents were recruited for participation. Groups of parents were randomly assigned by extension faculty members using a computer program (1:1:1 allocation) to three conditions: 168 parents to the online condition, 166 parents to the in-class condition, and 196 parents to the control condition (Fig. 1). Data from the groups randomly assigned to the online condition were examined in this paper. Because the analyses here employed about a third of the larger sample, and the intra-class correlation for videos accessed was higher than expected, a post hoc power analysis showed less power than the larger study (power of .80 to identify a medium effect size, *f* = .15).

The vast majority of participants in the online condition were female (95.9%): primarily mothers (90.5%) and grandmothers (4.8%). Mean participant age was 36.0 years (*SD* = 7.8). About half (54.1%) of the children were female with a mean age of 4.5 years (*SD* = 1.8). Families had a mean of 2.7 children (*SD* = 1.3) living in the home. Almost three-quarters of participants were Hispanic (72.8%). Race of participants was 92.3% White, 4.2% Black, 2.1% American Indian, and 1.4% Asian/Pacific Islander. Education ranged from less than sixth grade (12.3%) to college graduate (7.6%). The most common levels were high school graduate/GED (24.0%) and attended some college (22.6%). Only 27.6% were currently employed. Most were married (67.4%); 17.4% were never married and 15.2% widowed, separated, or divorced. Although participants were required to have smartphone access to participate, families varied in number of computers in the home: none (22.0%), one (49.0%) and two or more (29.0%).

### Description of the Intervention

As described in Hughes et al. (2020), parents were offered nine weekly ESBA, small group, in-person lessons (60–90 min each). Per EFNEP guidelines, classes were facilitated by peer educators from the local community. For parents in the online and in-class conditions, FFYF content was included starting on the third week (seven weeks total). Each week, parents in the *in-class* condition received the FFYF materials: a video, an interactive activity to reinforce the video content, and a printed infographic to take home summarizing the video content. Parents in the *online* condition received the FFYF content through text message links sent a few days after each ESBA lesson. The first text contained a link to the video viewed by the corresponding in-class group and a link to an online activity paralleling the in-class activity. Later that week, parents received a text with a link to an infographic and a reminder to complete the online tasks. Once the online materials were made available, facilitators instructed parents to complete the online tasks as part of their participation. A female, bilingual, online coordinator, introduced to the parents by video, sent all texts and was available throughout the study for parents needing assistance. Because classes of parents were randomly assigned to one of the three conditions, and the same facilitators taught the in-person classes in all three conditions, facilitators were trained to avoid mentioning FFYF content in the online and control classes. Fidelity observations showed that minimal cross-contamination occurred across the conditions (Aragón et al., 2021).

The seven FFYF content videos were edited versions of the SEEDS videos: shopping with your child, trying new foods, portion sizes, responsiveness to cues of hunger and fullness, mealtime routines, environmental influences on eating, and parent/child roles during feeding. Parents in the online condition viewed a short video by the online coordinator to describe the online process; this video was not included in the following analyses. The FFYF videos ranged in length from 2 to 9 min; online activities typically took 5 to 10 min. An online system recorded which videos parents accessed. However, the system did not record multiple views of the videos, nor how long each video was played. Given that we could not tell what parents were doing when the videos were on, we refer here to “videos accessed,” not “videos viewed.”

### Program Implementation

As described in Hughes et al. (2020), programs and assessments were conducted at neighborhood locations. After a group of parents completed the pre-test assessments, the group was randomly assigned to one of the three conditions. Parents in all conditions completed the same assessments at pretest, posttest, and 6- and 12-months. The current paper examined pretest and posttest data only. Parents completed consent forms before participating and received $30 at pretest and $40 at posttest. The IRBs at the three universities approved all procedures. Seventy-two implementations (i.e., groups of parents assigned to one of the conditions) were conducted across all conditions with a mean of 7.36 participants per implementation (*SD* = 2.95).

All participants in an implementation (regardless of condition) attended the same group assessment sessions. English and Spanish questionnaires were available. Research assistants, masked to condition, read questionnaires to parents needing assistance. For 25% of the in-person sessions (in-class and online groups), observers assessed fidelity (i.e., whether facilitators correctly completed each part of the curriculum). Mean fidelity was 84%. Deviations from fidelity were minor—most involved shortening activities to finish on time (Aragón et al., 2021).

### Measures

A subset of the parental questionnaires from the SEEDS evaluation (Hughes et al., 2021) was used to evaluate FFYF. All questionnaires had been successfully used with Spanish-speaking participants by Hughes et al. (2021). The measures (described in detail in Hughes et al., 2020) were: 1) a demographic questionnaire; 2) six scores from the Food Parenting Inventory (FPI—Power et al., 2019) based, in part, on a second order-factor analysis of the data from the Hughes et al. (2022) sample—encouraging consumption of new foods (alpha = .85), family meals (alpha = .72), child involvement in food preparation (alpha = .87), mealtime structure (alpha = .80), responsiveness to child fullness cues (alpha = .70), and pressure to eat (alpha = .61); 3) the two feeding dimensions scores from the Caregiver’s Feeding Style Questionnaire (Hughes et al., 2005)—demandingness (alpha = .86) and responsiveness (alphas = .74—.84), as well as the 4-level categorical feeding style variable (i.e., authoritative, authoritarian, indulgent, and uninvolved); and 4) four scores from the Feeding Knowledge Questionnaire (Hughes et al., 2021)—home efficacy (parental efficacy for promoting child healthy food consumption at home, alpha = .83), away efficacy (parental efficacy for promoting child healthy food consumption away from home, alpha = .75), best practices knowledge (knowledge of best-practices feeding based on program content, alpha = .75), and a dichotomous score (correct or incorrect) reflecting responses to a question about how many servings it takes for children to accept a new food. Acculturation was measured with the Bi-Dimensional Acculturation Scale (BAS) (Marin & Gamba, 1996). Based on language use, language proficiency, and electronic media use, subscales were calculated for the Spanish and English domains. Because the scores on the Spanish subscale showed limited variability, only the English subscale was used here (alpha = 0.97).

Three measures of program engagement were examined: 1) participation in the posttest evaluations (i.e., the final meeting of the in-class sessions); 2) number of in-person ESBA lessons (out of nine) the parent attended; and 3) number of FFYF videos (out of seven) the parent accessed. Correlations between these measures were: posttest and in-person, *r*(166) = .78, *p* < .001; posttest and videos, *r*(159) = .35, *p* < .001; in-person and videos, *r*(159) = .40, *p* < .001. Because the frequency that parents accessed the online components (i.e., videos, online activities, and infographics) were highly intercorrelated (correlations > .90), only videos accessed was used to assess online engagement. The 6- and 12-month assessment sessions were not used for assessing engagement because they were so far removed in time from the in-class sessions and did not, in our opinion, reflect engagement with the educational program itself.

### Data Analyses

Multilevel analyses (SPSS, version 25), using the Linear Mixed Models program for the engagement variables (attendance and videos accessed) and the Generalized Estimating Equations program for the dichotomous posttest participation variable, examined whether parental engagement varied by location (Colorado or Washington State) or language (English or Spanish) of the classes. Participants were nested within the 24 online implementations.

Two sets of analyses examined the prediction of parent engagement from the demographic variables with sufficient variability. Correlations assessed the simple bivariate associations for continuous variables. When both variables were dichotomous, the phi coefficient was computed. Linear mixed models and generalized estimating equations analyses examined the independent associations of the demographic predictors with participant engagement. To minimize the number of predictors, only demographic variables showing significant bivariate associations with engagement were included. Separate analyses for the Hispanic parents examined acculturation. Based on parents’ ethnicity (i.e., parent responses on the demographic questionnaire) and a median split on the English acculturation subscale, parents were assigned to three groups: non-Hispanics, high-acculturated Hispanics, and low-acculturated Hispanics. This variable examined engagement as a function of ethnicity/acculturation.

Linear mixed models and generalized estimating equations examined dosage effects of the number of videos accessed on the posttest outcomes, statistically controlling for pretest scores—a more powerful approach than repeated measures (Rausch et al., 2003). Given that the distribution of the number of videos accessed was not normal, a four-level “videos accessed” variable with relatively equal *n’s* was used as a predictor in the mixed models analyses: 0 videos (*n* = 21), 1 – 2 videos (*n* = 29), 3—5 videos (*n* = 31), and 6 – 7 videos (*n* = 46). The total *n* of 127 refers to parents in the online condition with both pre- and posttest data. Analyses were conducted for each outcome variable with three predictor variables and their interactions (dosage, language of classes, and location). Similar analyses compared differences in outcomes between the control group and parents in the online group who had not accessed any videos.

## Results

### Descriptive Data on Program Engagement

#### Posttest Participation

Of the 168 participants in the 24 groups randomly assigned to the online condition, 127 completed the posttest assessments (75.6%). The generalized estimating equations analysis showed that more parents in the Spanish language classes (84.9%) completed the posttest assessments than parents in the English classes (64.0%), Wald *X*^2^ (1) = 9.33, *p* < .01. The main effect of location and the location by language interaction were not significant.

#### ESBA Attendance

The mean number of in-person ESBA lessons attended by the 168 participants in the online FFYF condition was 6.26 of 9 lessons (70%) (*SD* = 3.15). One third (32.7%) of these participants attended all 9 lessons, 39.3% attended 6–8 lessons, 11.9% attended 2–5 lessons, 8.9% attended one lesson, and 7.1% attended no in-person lessons. Participants in the Spanish language classes attended more in-person lessons (*M* = 7.02, *SD* = 2.69) than participants in the English language classes (*M* = 5.32, *SD* = 3.43), *F*(1,164) = 12.05, *p* = .001. The location main effect and the location by language interaction were not significant.

#### Online Materials Accessed

The percentage of parents in the online condition accessing *a given video* declined over the first three weeks and then stabilized (*n* = 161—data were not available for 7 parents). The percentages of videos accessed by week were 59.5%, 50.6%, 41.1%, 44.0%, 42.9%, 41.7%, and 37.5% respectively. The average participant accessed 3.31 videos (47%) (*SD* = 2.62). The values for online activities and infographics were similar (activities—*M* = 3.34, *SD* = 2.62, infographics—*M* = 3.30 *SD* = 2.56). Thirty percent of the parents accessed one or no videos; thirty percent accessed all or all but one video. A significant language by location interaction, *F*(1,157) = 5.56, *p* < .05, showed that in Colorado, parents in the Spanish classes accessed more videos (*M* = 3.44, *SD* = 2.44) than in the English classes (*M* = 2.48, *SD* = 2.66); Washington—Spanish: *M* = 3.12, *SD* = 2.41; English: *M* = 4.11, *SD* = 2.90.

### Predictors of Program Engagement

Nine of the demographic variables had sufficient variability for analysis: child sex, child age, parent age, parent ethnicity, parent education, marital status, current employment, number of children, and number of computers in the home. Table 1 presents the bivariate associations between these variables and the measures of program engagement: completed posttest, number of ESBA lessons attended, and number of FFYF videos accessed. Older parents and parents of girls attended more lessons and were more likely to complete the posttest. Married parents attended more lessons; currently employed parents attended fewer. Finally, Hispanic parents accessed fewer videos, whereas parents with more computers accessed more videos.

**Table 1:** Bivariate associations between demographic variables and participant engagement (n = 168)

Engagement Variable	Child Sex (1 = female, 2 = male)	Child Age in Months	Parent Age in Years	Parent Ethnicity (1 = non-Hispanic, 2 = Hispanic)	Parent Education (7 point scale)	Marital Status (1 = not-married, 2 = married)	Currently Employed (1 = no, 2 = yes)	Number of Children Living in Home	Number of Computers in Home
Completed Posttest	-.21*^a^	.04	.20*	.02^a^	.09	.09^a^	-.07^a^	-.05	-.07
Number of ESBA Lessons Attended	-.28***	.02	.28***	.15+	-.10	.18*	-.24**	-.13	-.07
Number of FFYF Videos Accessed	-.03	.01	.08	-.26**	.10	.13	-.08	.00	.17*

Pearson correlations unless otherwise indicated. With the exception of child age, depending upon the variable, the number of missing cases ranged from 20 to 26 (see text)

^a^Phi coefficient

+*p* < .08; *p < .05; **p < .01; ***p < .001

The analyses examining the independent contributions of the predictors yielded similar results (Table 2): older parents and parents of girls attended more lessons and were more likely to complete the posttests, whereas currently employed parents attended fewer lessons. Hispanic parents accessed fewer videos than non-Hispanic parents. Two significant associations in the bivariate analyses became non-significant: marital status and number of computers in the home. This was likely a consequence of the significant negative association between being married and employment, *Φ* = -.29, *p* = .001, and fewer computers for Hispanic parents, *t*(142) = -2.72, *p* < .01, Hispanics, *M* = 0.97, *SD* = 0.69; non-Hispanics, *M* = 1.32, *SD* = 0.73.

**Table 2:** Mixed Models and Generalized Estimating Equations Analyses Predicting Participant Engagement from Demographic Variables

	Completed Posttest^a,c^			Number of ESBA Lessons Attended^a,d^			Number of FFYF Videos Accessed^b,d^		
Demographic Predictor	*B*	*SE*	Wald *X*^2^	*B*	*SE*	*F*	*B*	*SE*	*F*
Child Sex (1 = female, 2 = male)	-1.13	0.58	3.83*	-1.34	0.43	9.70**	-0.31	0.43	0.51
Parent Age in Years	0.11	0.05	4.69*	0.09	0.03	9.41**	0.03	0.03	1.18
Parent Ethnicity (1 = non-Hispanic, 2 = Hispanic)	-0.35	0.57	0.37	0.25	0.51	0.23	-1.84	0.51	12.91***
Marital Status (1 = not-married, 2 = married)	0.24	0.61	0.15	0.53	0.50	1.11	0.66	0.50	1.73
Currently Employed (1 = no, 2 = yes)	-0.42	0.58	0.53	-1.11	0.50	4.95*	-0.61	0.50	1.48
Number of Computers in Home	-0.62	0.39	2.51	-0.34	0.31	1.18	0.19	0.31	0.38

^a^*N* = 142

^b^*N* = 138

^c^Generalized estimating equations analyses

^d^Mixed models analyses

*p ≤ .05; **p < .01; ***p < .001

Further analyses showed that the number of lessons attended, *F*(2,144) = 3.20, *p* < .05, and the number of videos accessed, *F*(2,140) = 7.12, *p* = .001, varied by acculturation. Low-acculturated Hispanic parents attended significantly more lessons (*M* = 7.55, *SD* = 2.46) than non-Hispanic parents (*M* = 6.18, *SD* = 2.92) and high-acculturated Hispanic parents (*M* = 6.62, *SD* = 2.83). The number of videos accessed significantly differed between all three groups: non-Hispanic parents accessed the greatest number (*M* = 4.68, *SD* = 2.81), low-acculturated Hispanic parents were second (*M* = 3.61, *SD* = 2.41), and high-acculturated Hispanic parents accessed the fewest (*M* = 2.65, *SD* = 2.34). Completion of posttests did not vary by acculturation, Wald *X*^2^(2) = 0.54, *n.s.* When these analyses were rerun controlling for the number of computers in the home (non-Hispanic parents reported a significantly greater number of computers than high acculturated or low acculturated Hispanic parents), the acculturation effect was still significant, *F*(2,136) = 6.54, *p* < .003, and the differences between the three groups remained.

### Dosage Effects

Because parents in the four video groups differed on marital status and ethnicity (married parents accessed more videos than unmarried parents, *X*^2^(3) = 7.62, *p* = .05, and Hispanic parents accessed fewer videos than non-Hispanic parents, *X*^2^(3) = 12.37, *p* < .01), these variables were controlled for in the dosage analyses. The videos accessed main effect was significant for 5 of the 13 outcome variables: encourage consumption of new foods, *F*(3, 108) = 4.00, *p* = .01; child involvement in food preparation, *F*(3, 108) = 4.09, *p* < .01; responsiveness (CFSQ), *F*(3,109) = 3.41, *p* < .05; best practice feeding knowledge, *F*(3, 109) = 5.74, *p* = .001; and knowledge regarding presentation of new foods to encourage acceptance, Wald *X*^*2*^ (1) = 11.79, *p* < .001. The “location by videos accessed” interaction was also significant for the new foods variable, Wald *X*^*2*^ (1) = 5.53, *p* < .05. The videos accessed variable significantly predicted this outcome for parents in Washington, Wald *X*^*2*^ (1) = 11.91, *p* < .001, but not Colorado, Wald *X*^*2*^ (1) = 0.80, *n.s.*

**Fig. 2:**
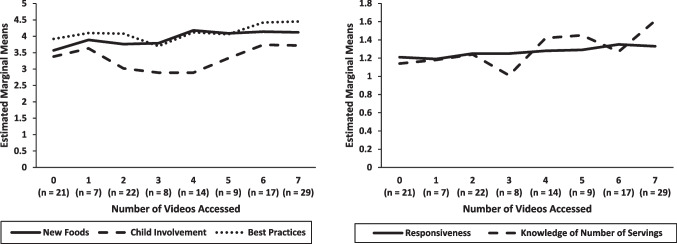
Program outcomes for participants in the online condition as a function of number of FFYF videos accessed (Estimated Marginal Means on Outcome Variables Controlling for Pretest Scores, Parent Ethnicity, and Marital Status)

Figure 2a and b show that for most variables, parents showed better outcomes with increased video exposure. The figures present the estimated marginal means on the outcome variables that varied as a function of video exposure (controlling for pretest scores, parent ethnicity, and marital status). Although the small *n’s* for the number of videos accessed required combining various levels for the analyses described above (i.e., less than 10 parents accessed 1, 3, or 5 videos), the values in the figures are reported separately (eight values ranging from 0–7 videos) to demonstrate how outcomes varied by the number of videos accessed. Only CFSQ responsiveness showed a simple linear increase with the number of videos accessed. For the other variables (except child involvement in food preparation), the outcome variables were mostly stable from zero to three videos accessed, followed by an increase after the third video. Child involvement in food preparation showed a curvilinear relationship, with the highest levels for parents accessing 0, 1, 6, or 7 videos. For all except child involvement, parents in the highest exposure category (6–7 videos) showed significantly higher values (*p* < .05) on the outcome variables than parents in the first two categories (0–2 videos). Parents in the first two categories (0 versus 1–2 videos accessed) did not differ from each other. With the exception of child involvement, parents in the third category (3–5 videos accessed) showed intermediate values.

Finally, follow-up analyses examined whether the means for the outcome variables for parents in the online condition who accessed no videos differed significantly from the means for parents who had been randomly assigned to the control group and had no opportunity to receive FFYF content. Analyses for the five outcomes showed no differences between the two groups.

## Discussion

Together, this study provides some “lessons learned” that should be helpful for the development and implementation of online parenting interventions for families with low incomes. In this study, despite the high levels of in-person attendance in the ESBA classes (the mean participant attended 70% of the lessons), parents in the online condition accessed only about half of the videos (*M* = 47%). This percentage is similar to the percentages reported by Love et al. (2016) (36% and 51%) and Rojas et al. (2021) (45%), but lower than the value reported by Estrada et al. (2019) (74%). The degree to which parents accessed videos in the current study declined over the first three weeks, with about 60% of participants accessing the first video and about 40% accessing a given video from the third video on. This shows that even among a highly motivated group of parents who regularly attended in-person classes, the average parent was not exposed to half of the online content. This points to the importance of building repetition into online interventions to ensure that parents get exposed to the core content of the program. If an individual video presents unique information not covered in subsequent videos or online materials, one would expect less than half of the parents to receive that content.

A second lesson is that program effects can vary significantly as a function of the amount of online content accessed. In the current study we found significant dosage effects for five outcomes—all variables showing significant online condition effects in the RCT analyses reported in Hughes et al. (2022). However, dosage effects for these variables did not typically occur unless parents had accessed more than half the videos. Two additional outcomes (pressure to eat and mealtime structure) showed online effects in Hughes et al. (2022), but these effects were only significant for the English language classes. The dosage analyses presented here were likely underpowered to identify the moderating effects of program language. Although causal conclusions cannot be drawn from dosage analyses (unless parents are randomly assigned to dosages), the finding that parents in the online condition who accessed no videos did not differ from control parents on the outcome variables is consistent with a causal interpretation.

Third, the analyses predicting individual differences in engagement are consistent with the argument that online delivery can help address some of the barriers to in-person participation for parents with low incomes (Corralejo & Rodriguez, 2018). In this study, currently employed parents were less likely to attend ESBA lessons possibly due to work conflicts, transportation issues, or lack of childcare. However, employed and unemployed parents did not differ on the number of videos accessed, possibly due to the flexibility provided through online participation.

Analyses of ethnic differences in engagement suggested that the online option may not be optimal for some Hispanic parents. Parents in the Spanish speaking classes showed greater ESBA lesson attendance than parents in the English-speaking classes (78% versus 59%), with low-acculturated Hispanic parents showing the highest in-person attendance. Additionally, the number of videos accessed was highest for non-Hispanic parents, with highly-acculturated Hispanic parents viewing the fewest. The higher level of online involvement among less acculturated Hispanic parents is consistent with Perrino et al. (2018) who found higher levels of online engagement among low-acculturated Hispanic parents. This was true for both in-person and online engagement in the current study, although, for low-acculturated Hispanic parents, the level of in-person engagement (84%) was considerably higher than online engagement (52%). The ethnic and acculturation differences found here may reflect the importance of in-person interaction for less acculturated Hispanics (*personalismo*) (Delgado, 1995), particularly when peer educators facilitate the classes. Moreover, in-person classes may be more important for less acculturated Hispanic parents because as likely recent immigrants to the U.S., they may have wanted to interact with other Spanish-speaking parents in an in-person setting. For these parents, in-person classes may play an important social function as well. Future research should examine ways to engage highly acculturated Hispanic parents in both in-person and online settings.

Finally, the findings suggest that it is important to find ways to engage parents of boys and younger parents in such online interventions. Although previous findings show inconsistent associations between sex of the child and in-person parent engagement (Robinson et al., 2016), the finding that parents of girls attended more in-person lessons in this study might reflect the possibility that parents may have been more concerned about the weight status of their girls than their boys (Keller et al., 2019). The finding of lower attendance in this study for younger parents is consistent with parenting interventions in other domains (Robinson et al., 2016).

The current study had several limitations: it was underpowered to identify moderators of dosage effects, the outcome assessments were parent-report measures, and some ethnic/racial groups were not well-represented. Moreover, because participants were paid for attending the assessment sessions, it is not clear whether the same levels and predictors of engagement would have occurred in the absence of incentives. Finally, due to the research design, facilitators were not allowed to talk about the online content during the in-person sessions. Higher levels of online engagement might have occurred if facilitators had encouraged online viewing of the materials. Despite these limitations, the study had many strengths: the examination of participant engagement in an existing, nationwide intervention; the assessment of both in-person and online engagement; and the use of well-validated measures for the dosage analyses. Future studies would benefit from employing mixed-methods approaches (e.g., adding qualitative assessments to inform the quantitative results) or simultaneously examining patterns of engagement across multiple assessments (e.g., Lin & Masse, 2021). Given the low levels of online engagement found here, future studies should identify strategies that might increase engagement (e.g., individualized content, feedback, or progress monitoring) and examine how combinations of online and in-person strategies may promote engagement in various populations and domains.

## Data Availability

The data that support the findings of this study can be made available upon reasonable request and a data use agreement may be required.
